# A Narrative Review of Bertolotti's Syndrome: Etiology, Classification, Diagnosis, and Treatment

**DOI:** 10.7759/cureus.109205

**Published:** 2026-05-19

**Authors:** Matyas Abel Tsegaye, Andrea Muffly

**Affiliations:** 1 Biomedical Sciences, Bertolotti's Syndrome Foundation, Chicago, USA; 2 College of Health Science, Old Dominion University, Norfolk, USA; 3 Occupational Health, Bertolotti's Syndrome Foundation, Chicago, USA

**Keywords:** bertolotti's syndrome, castellvi classification, chronic low back pain, jenkins classification, lumbosacral transitional vertebra, neuropathic pain

## Abstract

Bertolotti's syndrome is a congenital condition characterized by chronic low back pain stemming from a lumbosacral transitional vertebra (LSTV). Because the condition is frequently overlooked, patients often face years of delayed diagnosis before the true source of their pain is identified. Furthermore, current information regarding the biological origins and clinical management of the condition remain scattered. To address this knowledge gap, this narrative review aims to provide a comprehensive overview of Bertolotti’s syndrome by consolidating these disparate sources and suggesting new connections. We synthesize the latest understanding of this condition, ranging from its genetic origins to modern clinical management. We examine the biomechanical alterations caused by an LSTV and discuss the advantages of Jenkins classification over Castellvi classification. The diagnostic protocol is presented as a multi-step process, emphasizing the necessity of fluoroscopy-guided diagnostic injections to distinguish incidental findings from true pathology. Finally, we present the consensus treatment protocol based on the latest findings and identify critical gaps in the literature regarding standardized care protocols, unified clinical nomenclature, and long-term surgical outcomes.

## Introduction and background

Bertolotti's syndrome (BSy) represents a complex and often underdiagnosed cause of chronic low back pain, originating from a congenital anomaly at the lumbosacral junction [1]. First described in 1917 by Italian surgeon Mario Bertolotti, the syndrome establishes a clinical link between a specific anatomical variant called the lumbosacral transitional vertebra (LSTV) and the onset of chronic low back pain [2]. 

Several studies have tried to ascertain the incidence rate for LSTV in the general population. These studies show a large variance ranging between 4-36% of people having LSTV across different studies [3,4]. The most accurate figure on the incidence of LSTV in the general population was found to be close to 20% after analysis of radiological images of 6200 individuals [5]. Although LSTV is present in nearly one in five people, we do not observe a similar number of people diagnosed with BSy [6]. 

Despite the high prevalence of LSTV in the general population, there remains a lack of comprehensive resources that bridge the gap between the biological origins of the condition and its clinical management. This review aims to address this deficit by integrating the genetic basis of BSy with the latest consensus on diagnostic protocols and treatment options. We provide a high-level overview of the most recent findings that provide the latest understanding of the syndrome's complexity beyond simple anatomical classification. Furthermore, we identify critical areas where further research is needed, particularly regarding the standardization of conservative care and the evaluation of long-term surgical outcomes, to better inform future therapeutic strategies.

## Review

Methodology

A comprehensive search of the literature was conducted using the PubMed and Google Scholar databases, covering the period from the publication of the seminal paper by Antonio Castellvi in 1984 [7] through November 2025. 

Inclusion criteria were peer-reviewed systematic reviews, meta-analyses, cohort studies, and case reports, written in English, that addressed pathophysiology, genetic origins (including HOX gene patterning defects), diagnostic protocols, or clinical management of BSy. Exclusion criteria were articles not published in English and articles published before 1984.

The search strategy utilized the following keyword string: ("Bertolotti’s Syndrome" OR "Bertolotti Syndrome") and ("lumbosacral transitional vertebra" OR "LSTV"). Related congenital conditions were also manually searched to ensure a broad capture of relevant pathophysiology. To supplement the electronic search, "snowballing" methods were used, where the reference lists of retrieved articles were manually screened for further review.

Articles focusing on lumbosacral anatomy in asymptomatic populations were specifically included to evaluate the incidence rate of LSTV in the general population. The selection and data extraction process was performed by the corresponding author, focusing on biological foundations, biomechanics, diagnostic imaging (X-ray, MRI, single-photon emission computed tomography (SPECT)/CT), and the standard treatment cascade.

No formal risk of bias assessment of individual studies was performed due to the narrative nature of this synthesis. No quantitative synthesis was reported due to heterogeneity of included studies; instead, articles were evaluated using a thematic synthesis approach to integrate findings, identify clinical consensus, and highlight current gaps in the literature.

LSTV classification

The modern classification of LSTV anatomy was first introduced by Antonio Castellvi to characterize the different types of transitional anatomy observed in patients [7]. Castellvi categorizes LSTV anatomy into four types [7].

Type I

Type I signifies an enlarged transverse process that does not create a pseudoarticulation with the sacrum. It is subcategorized based on whether the enlarged transverse process is unilateral (Type IA) or bilateral (Type IB). 

Type II

Type II is an LSTV anatomy where the enlarged transverse process creates a pseudoarticulation with the sacrum. Type IIA indicates that only one transverse process is enlarged to form a pseudoarticulation with the sacrum. It is categorized as Type IIB if both transverse processes create a pseudoarticulation with the sacrum.

Type III

Type III identifies a complete fusion of the transverse process with the sacrum. Type IIIA indicates that the transverse process is fused to the sacrum on one side whereas Type IIIB indicates that the transverse process is fused bilaterally.

Type IV

Type IV refers to a mixed anatomy where one side is Type IIA (i.e. transverse process forms a pseudoarticulation) and Type IIIA (i.e. complete fusion of transverse process with sacrum).

Despite its broad adoption since its inception in 1984, the Castellvi classification system has two major limitations. First, the classification was developed to identify lumbosacral disc herniations in patients. It does not consider the role of LSTV in causing BSy. Second, it fails to consider the contact of the enlarged transverse process to the iliac crest. 

In 2023, Dr. Arthur Jenkins and his team addressed both limitations in the Castellvi classification by proposing a revised system termed the Jenkins classification [8]. Although it is not broadly adopted yet, we consider this classification to be a significant improvement over the Castellvi classification. We expect that these advantages will lead to the Jenkins classification to gain broader adoption over time. The Jenkins classification also categorizes LSTV into four types.

Type 1

**Figure 1:**
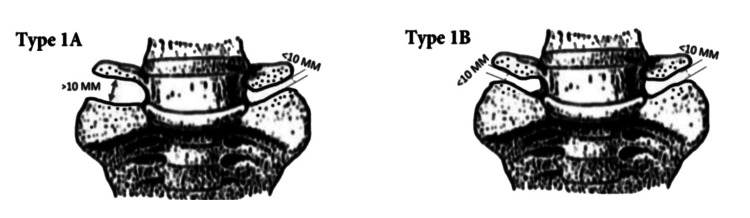
Type 1 lumbosacral transitional vertebra (LSTV) based on Jenkins classification: (left) Type 1A and (right) Type 1B Copyright/license: This figure has been reproduced from Jenkins et al. 2023 [8], which is an open-access article distributed under the terms and conditions of the CC BY-NC-ND 4.0 license. Additional permission for reuse was obtained from the corresponding author.

Type 1A: Enlarged transverse process on the last lumbar segment with less than 10mm gap, but over 2mm gap, between the transverse process and sacral ala on one side only [8]. The other side has a “normal” distance of over 10mm (Figure 1) [8].

Type 1B: Both transverse processes of the last lumbar segment have less than 10mm gap, but over 2mm gap, between the transverse process and sacral ala (Figure 1) [8].

Type 2

**Figure 2:**
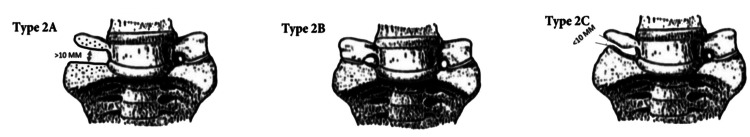
Type 2 lumbosacral transitional vertebra (LSTV) based on Jenkins classification: (left) Type 2A, (middle) Type 2B, (right) Type 2C Copyright/license: This figure has been reproduced from Jenkins et al. 2023 [8], which is an open-access article distributed under the terms and conditions of the CC BY-NC-ND 4.0 license. Additional permission for reuse was obtained from the corresponding author.

Type 2A: Incomplete lumbarization/sacralization with enlarged transverse process that creates a pseudo-articulation with itself and the sacrum (less than 2mm distance) on one side only [8]. The other side has a “normal” distance of above 10mm (Figure 2) [8]. 

Type 2B: Symmetrical anatomy where both transverse processes have a pseudo-articulation on both sides with the sacrum with less than 2mm distance (Figure 2) [8].

Type 2C: Hybrid anatomy where one transverse process has Type II anatomy (i.e., forms pseudo-articulation with the sacrum) and the other transverse process has Type IA anatomy (i.e., has between 2mm to 10mm distance between itself and the sacrum) (Figure 2) [8].

Type 3

**Figure 3:**
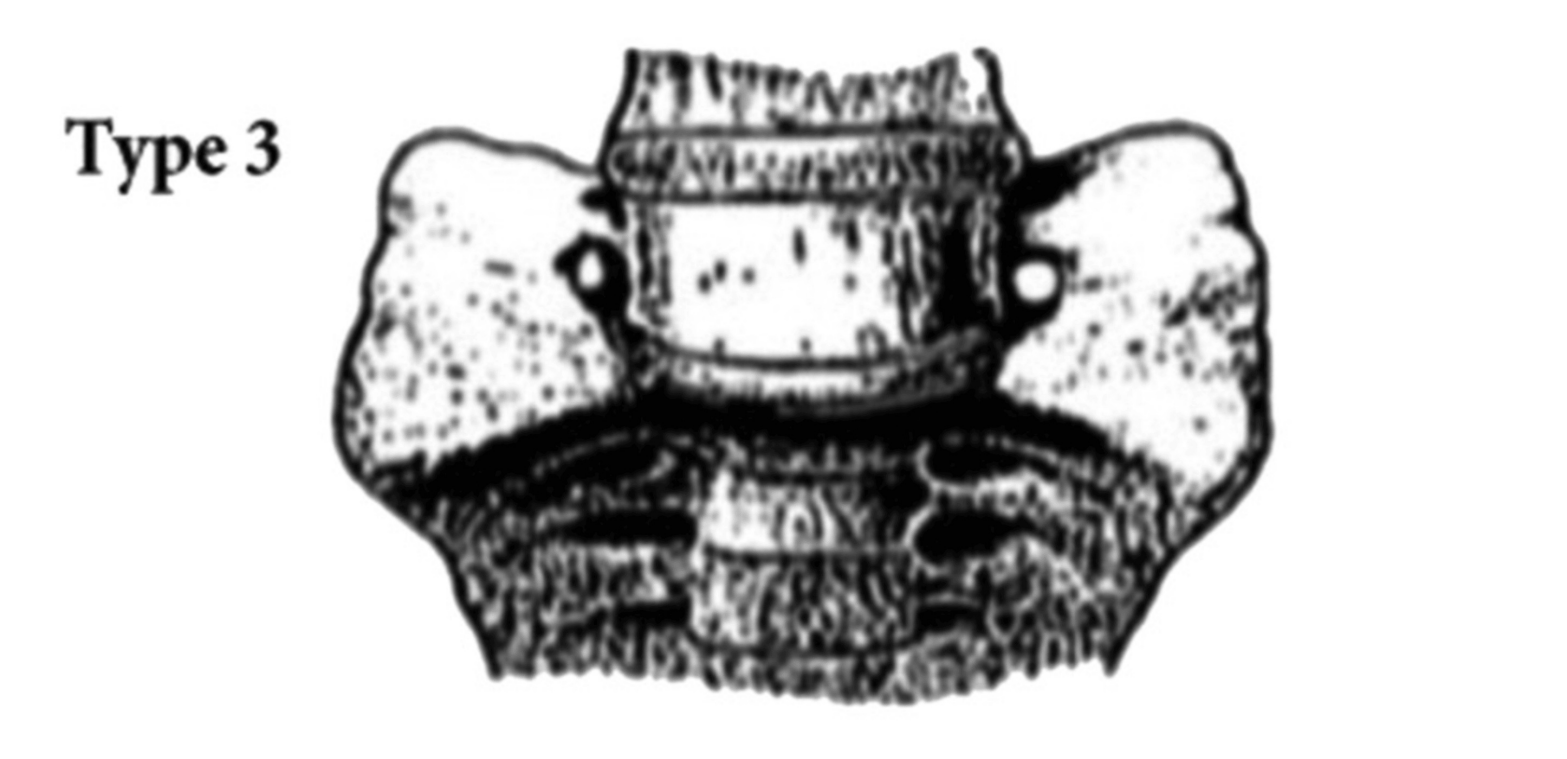
Type 3 lumbosacral transitional vertebra (LSTV) based on Jenkins classification Copyright/license: This figure has been reproduced from Jenkins et al. 2023 [8], which is an open-access article distributed under the terms and conditions of the CC BY-NC-ND 4.0 license. Additional permission for reuse was obtained from the corresponding author.

Bilateral lumbarization/sacralization with complete osseous (bone) fusion of the transverse processes to the sacrum (Figure 3) [8].

Type 4

**Figure 4:**
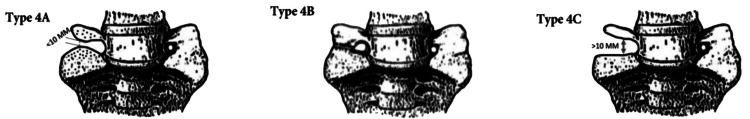
Type 4 lumbosacral transitional vertebra (LSTV) based on Jenkins classification: (left) Type 4A, (middle) Type 4B, (right) Type 4C Copyright/license: This figure has been reproduced from Jenkins et al. 2023 [8], which is an open-access article distributed under the terms and conditions of the CC BY-NC-ND 4.0 license. Additional permission for reuse was obtained from the corresponding author.

Type 4A: Lumbarization/sacralization with complete osseous fusion of the transverse process to the sacrum on one side only [8]. The other side has Type I anatomy with a transverse process at a distance of less than 10mm from the sacrum (Figure 4) [8].

Type 4B: Lumbarization/sacralization with complete osseous fusion (i.e., Type 3-like anatomy) on one side and an incomplete lumbarization/sacralization of the transverse process on the other side (i.e., Type 2-like anatomy) (Figure 4) [8]. 

Type 4C: Lumbarization/sacralization with complete osseous fusion of transverse process to the sacrum on one side only [8]. The other side has “normal” anatomy with a transverse process at a distance of more than 10mm from the sacrum (Figure 4) [8].

**Figure 5:**
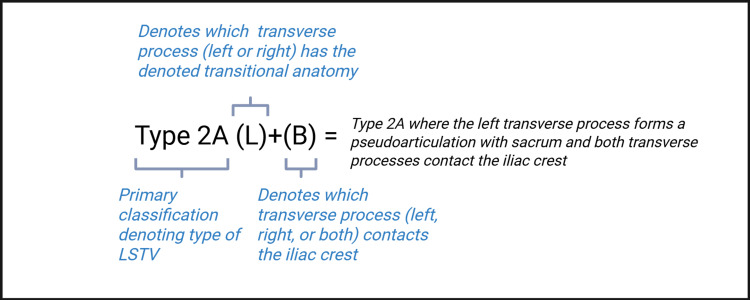
How to designate lumbosacral transitional vertebra (LSTV) anatomy using Jenkins classification Image created by authors using BioRender.com.

The Jenkins classification also identifies the laterality of the enlarged transverse process by using L/R designations. For example, Type 1A (L) denotes the left enlarged transverse process has a <10mm gap with the sacrum whereas the right side has >10mm gap. There is also a secondary classification denoted by adding +L/R/B to the primary classification to determine any contact between the transverse process and the iliac crest. For example, Type 2A (L)+(B) refers to both transverse processes contacting the iliac crest (Figure 5). 

Lumbarization Versus Sacralization of LSTV

LSTVs may be described as lumbarized (i.e., lumbarization of the uppermost sacral segment) or sacralized (i.e., sacralization of the lowest lumbar vertebral body) [9].

Lumbarization is a spinal abnormality where the first sacral vertebra is not fused with the rest of the sacrum, which causes the appearance of six lumbar-type vertebrae and only four sacral vertebrae [10]. Such transitional anatomy can present with clinical symptoms including painful movement and difficulties performing activities of daily living (ADLs) [10,11]. 

Sacralization is a common spinal abnormality creating transitional lumbosacral anatomy where L5 is partially or fully fused to sacral ala and is referred to incomplete sacralization or complete sacralization accordingly [12]. Sacralization predisposes the spine to be more susceptible to degenerative changes due to the altered lumbosacral load-bearing spinal mechanics [12].

Biomechanical changes due to Bertolotti’s syndrome

The presence of LSTV fundamentally alters the biomechanics of the lumbosacral spine, initiating a cascade of pathological changes that can lead to pain [2,13]. In a normal spine, forces are distributed through the mobile lumbar segments to the stable sacropelvic complex. However, an LSTV introduces hypomobility and greater forces being applied to the lumbar segment immediately above it [2]. 

The altered biomechanics can result in patients presenting with lower back pain. The source of this pain can be attributed to various factors stemming from the changes caused by the LSTV. Some of these sources of pain include the following.

Direct Effect of LSTV

Arthritis of the pseudo-articulation: In LSTV types where the lumbar transverse process forms a “false joint” with the sacrum (i.e., Type 2 and Type 4), the bone-on-bone contact leads to mechanical grinding, inflammation, and the formation of osteophytes within the false joint [2,8]. This can lead to direct localized, aching pain. 

Radiculopathy: LSTV can cause radiculopathy which can include muscle weakness, hypoesthesia (decreased sensation), numbness, and tingling [14-16]. It is important to note that not all these symptoms will be uniformly present in patients. For instance, a case report by Kapetanakis et al. shows how the patient did not have muscle weakness or diminished reflexes [16].

Radiculopathy can be caused by direct compression of the exiting L5 nerve root due to the enlarged transverse process. Alternatively, the local inflammation caused by osteophyte formation due to pseudo-articulation can lead to irritation of the L5 nerve root. Alternatively, the hypermobility at the L4-L5 level due to LSTV can cause disc herniation at that level [15]. 

Secondary Effects of LSTV

Myofascial pain: The body’s attempt to stabilize the anomalous anatomy can cause increased strain on the musculature around the LSTV. This can lead to hypertonicity in the quadratus lumborum (QL) and the Iliopsoas that can cause formation of chronic strain and trigger points in these muscles [1]. 

Contralateral facet joint stress: The asymmetrical motion caused by unilateral LSTV can place excessive load on the facet joint on the opposite side of the pseudo-articulation. The spinal load imbalance caused by the unilateral LSTV can lead to facet joint pain and development of osteoarthritis of the facet joint [15].

Adjacent segment disease: Compensatory hypermobility of the segment above the LSTV can lead to acceleration of degeneration of the disc [2,3]. This, in turn, leads to higher incidence of disc herniation, annular tears (i.e., tear of the outer wall of the disc), and spinal stenosis compared to individuals with typical anatomy [3].

Genetic basis for Bertolotti’s syndrome

BSy is a congenital condition that occurs due to an error in the early embryonic stages of development [17]. The family of genes involved in determining the segmentation of the spine segments is the HOX gene family. These genes are a family of transcription factors that determine whether each vertebra becomes a cervical, thoracic, lumbar, or sacral segment [18,19]. 

**Figure 6:**
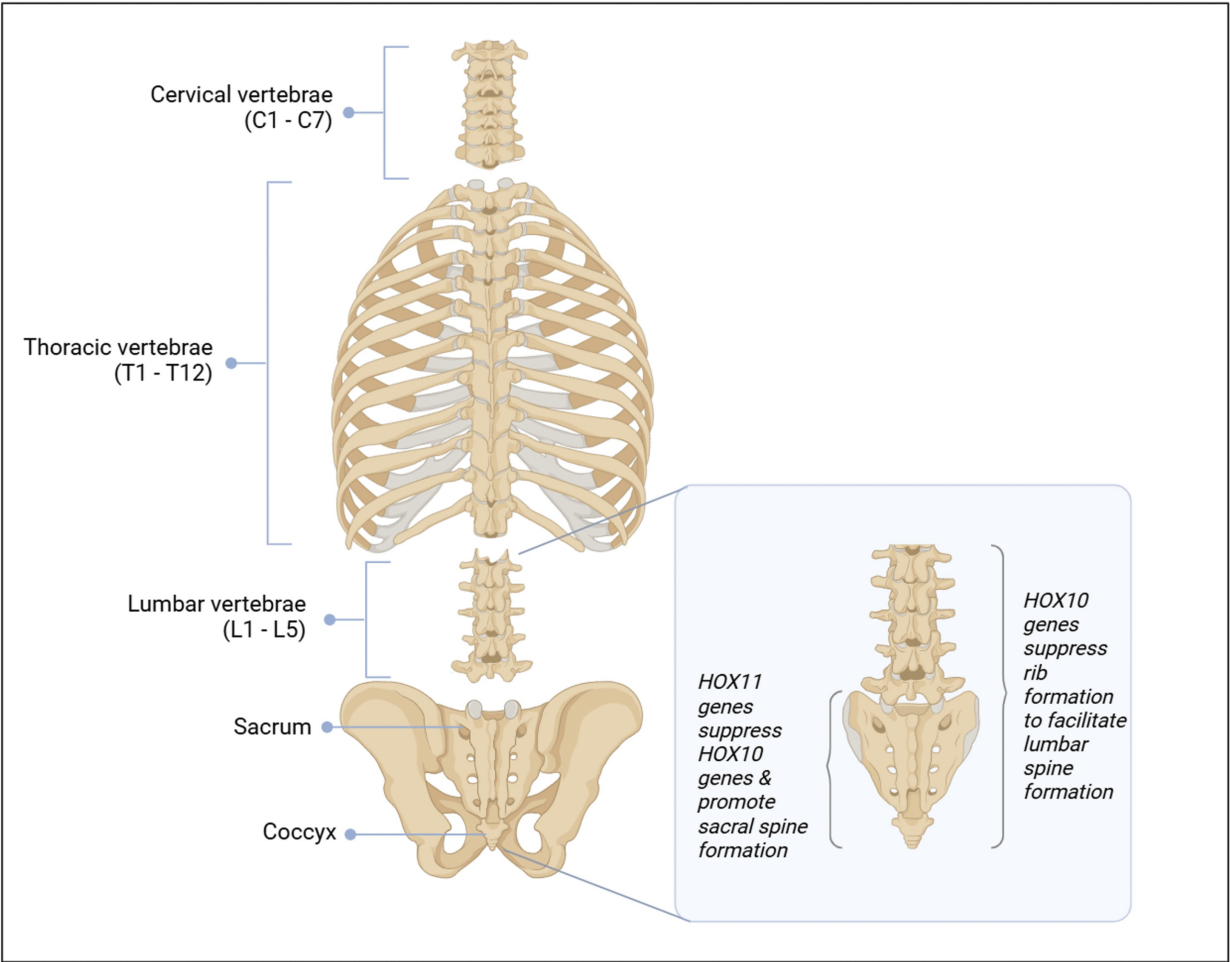
Human spine segments and role of Hox10 and HOX11 in lumbosacral spine development Image created by authors using BioRender.com.

There is a strong indication that the development of a LSTV, the basis for BSy, is a consequence of a developmental patterning defect [17,8,20]. HOX10 genes confer the development of non-rib-bearing vertebrae, i.e., lumbar vertebral segments, whereas HOX11 genes are responsible for the development of the sacrum (Figure 6) [21]. 

Taken together, several studies suggest that LSTV results from a boundary shift in the expression pattern of HOX10 and HOX11 gene groups [22]. Cranial shift of the expression of HOX11 genes may result in the partial or complete sacralization of the last lumbar segment [22]. Conversely, caudal shift of the expression of HOX10 genes can confer partial or complete lumbarization at the S1 level [22]. 

Congenital conditions observed in patients with Bertolotti’s syndrome

Certain congenital conditions have been observed in a subset of patients diagnosed with BSy. Based on currently available literature, the co-incidence of these congenital conditions and BSy is purely correlative; neither causative links nor commonalities in the genetic changes leading to these conditions have been found. Further investigation is warranted to better characterize the prevalence and potential comorbidities of these conditions. However, there is indication that the presence of transitional anatomy at the lumbosacral segment means there is a higher likelihood of finding transitional anatomy (e.g., thoracolumbar, or cervicothoracic) at other levels [15]. Some of the conditions that have been observed in patients are shown below:

Hypermobility Ehlers-Danlos Syndrome (hEDS)

hEDS is a rare heritable tissue disorder that causes general joint hypermobility [23]. It is the most common type of EDS, accounting for about 90% of cases. Some patients with hEDS have also been shown to have craniocervical instability (CCI) and thoracic outlet syndrome (TOS) [23]. There is currently no laboratory test that can definitively identify patients with hEDS [23].

Spina Bifida Occulta (SBO)

SBO is a rare congenital disorder where there is incomplete neural tube closure during fetal development in the lumbosacral region [24,25]. SBO is a mild version of spina bifida where a gap is present between the vertebrae, but the meninges and spinal cord remain within the vertebral canal [24,25].

Cervical Stenosis

Cervical stenosis is caused by the narrowing of the spinal canal in the cervical spine [26]. Cervical stenosis can be congenital or acquired due to degenerative changes after birth [26]. 

Extra L6 Vertebra

There are two ways through which an L6 vertebra can form. First, incorrect spine segmentation during fetal development determined by the HOX gene family can lead to lumbarization of the first sacral segment (S1) or the lumbarization of the last thoracic segment (T12) [21,27]. Second, patients can have a true L6 vertebra where they have 25 vertebrae versus the normal 24 [28]. We have not found a consensus in the literature on how this anatomy impacts stability of the lumbosacral region or other clinical conditions associated with having an L6 vertebra.

Thoracic Outlet Syndrome 

TOS describes a set of symptoms in the neck and shoulder characterized by numbness, tingling, and pain caused by the compression of the nerves and blood vessels traversing the narrow space between the clavicle and the first rib [29]. Neurogenic TOS, caused by the compression of the C5-T1 brachial plexus nerve roots, accounts for over 90% of TOS cases [30]. Furthermore, patients with a cervical rib (i.e., a transitional anatomy) are predisposed to developing neurogenic TOS [29].

Diagnosis of Bertolotti’s syndrome

**Figure 7:**
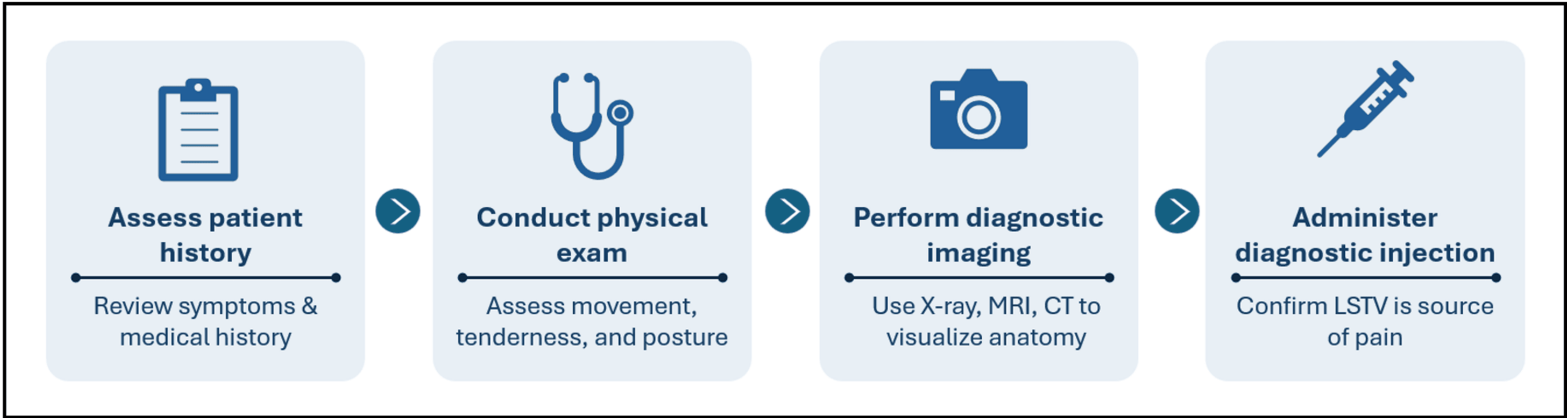
Diagnostic protocol for Bertolotti's syndrome Image created by authors using PowerPoint (Microsoft, Redmond, WA, USA). LSTV: lumbosacral transitional vertebra

Clinical presentation of pain symptoms in patients with BSy is not always straightforward. Therefore, the diagnosis of BSy is a multi-step process that requires a critical review of patient history, thorough physical evaluation, precise imaging, and administration of a diagnostic injection (Figure 7).

Assess Patient History 

Patients with BSy tend to present with chronic lower back pain that started early in life [1,11,31]. However, this is not always the case. Given the lack of consistent triggers for symptom onset, patient history alone cannot provide sufficient information to make a diagnosis. 

Conduct Physical Exam

Physical exam can reveal localized tenderness around the posterior superior iliac spine (PSIS), the SI joint, and the paraspinal muscles [6]. Furthermore, individuals may present with limited lumbar range of motion as well as positive tests on the FABER and Gaenslen tests [2,6]. 

However, patients with BSy are unique as they present with low back pain, but may present with a normal physical examination [32]. Given the non-specificity of a physical exam in diagnosing BSy, it is important that it is coupled with the appropriate radiological imaging and diagnostic injections. 

Diagnostic Imaging

Diagnostic imaging is a critical component in the diagnostic protocol for BSy. It is used to identify the presence and type of LSTV. Furthermore, it helps identify other clinical conditions that could be contributing to the patient’s symptoms. The imaging modalities utilized are as follows.

**Figure 8:**
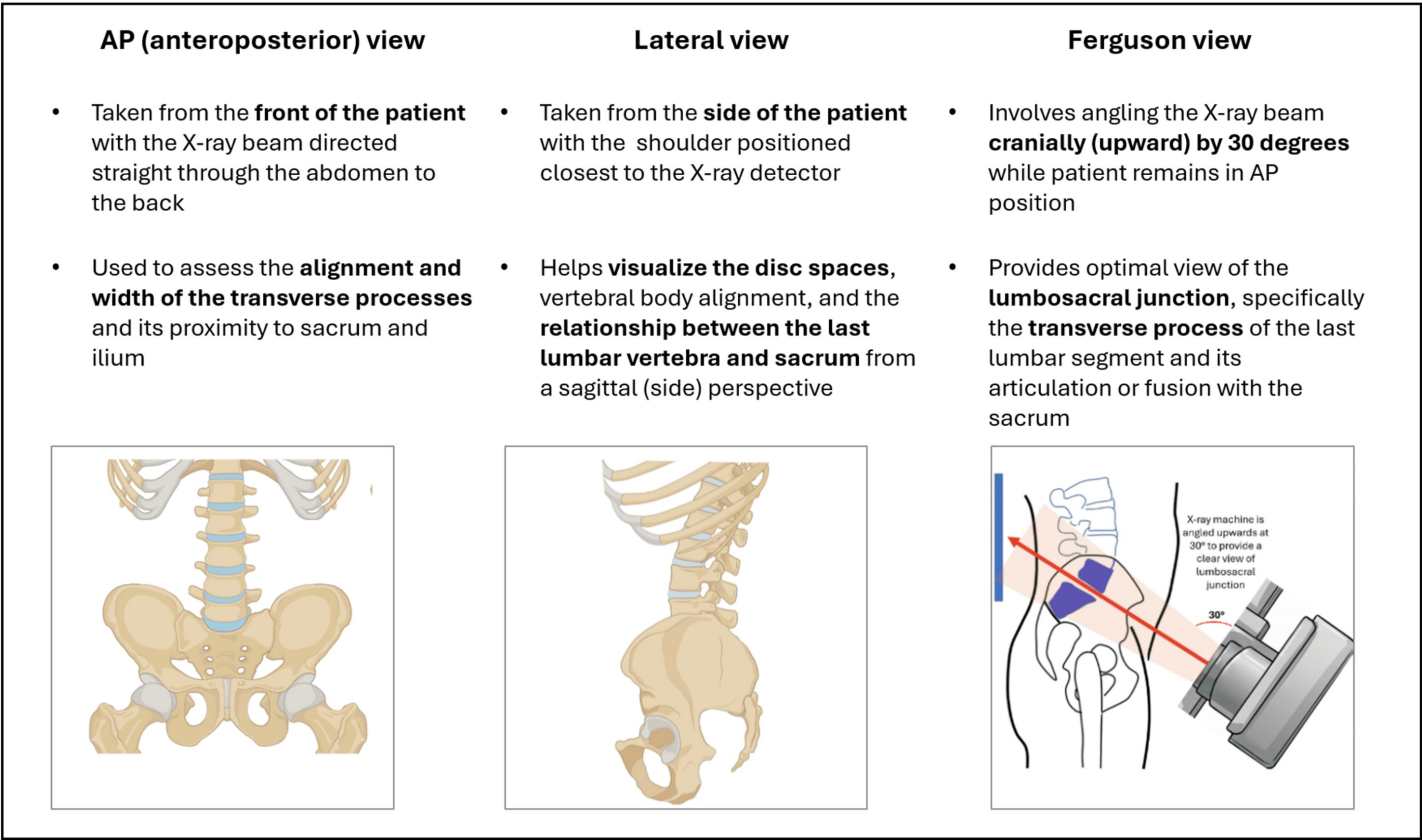
Recommended X-ray imaging views Image created by authors using BioRender.com

X-ray: Clear visualization of the lumbosacral region for the purpose of identifying LSTV requires a combination of three imaging views and three positional postures: anteroposterior (AP), lateral, and Ferguson view (Figure 8) [9]. The Ferguson view is the most important imaging view as it provides an unobstructed view of the lumbosacral junction [15]. Critically, the presence of LSTV often correlates with higher likelihood of similar anomalies being present at other levels [15]. X-ray imaging of the entire spine can address these concerns. It ensures an accurate count of vertebrae in each segment, determines whether the LSTV is due to lumbarization of S1 or sacralization of L5, and assesses whether other vertebral anomalies such as a transitional anatomy are present at other levels. 

Magnetic resonance imaging: MRI goes beyond identifying the presence of LSTV. It helps to clearly evaluate soft tissue in the lumbosacral region. It helps identify any comorbidities that could be the source of the patient’s pain such as disc herniation, annular tear, facet joint arthrosis, and nerve root compression [2]. 

Computed tomography scan: This is considered to be the best method to visualize LSTV. However, it is not the first-line imaging technique for assessing the presence of LSTV since it exposes patients to higher levels of radiation [9]. The Ferguson view provides enough clarity of the lumbosacral junction to assess the LSTV classification of a patient. CT images are used to develop a 3D model of the lumbosacral region which can, in turn, aid in the devising appropriate surgical approach. 

SPECT/CT scan: In certain cases, SPECT/CT scans are used to locate the site of pain. This technique uses a radioactive tracer that localizes to sites of osteoblast activity which localizes to the sites of arthrosis formed due to the pseudo-articulation formed by LSTV [33]. It is important to note that patients with BSy may not always have a positive signal on SPECT/CT scan. A negative SPECT/CT scan result does not rule out LSTV as the pain generator [34]. This must be confirmed using diagnostic injection.

Diagnostic Injection 

The presence of LSTV does not necessarily mean that it is the pain generator. The final and most critical step is a diagnostic injection of local anesthetic (e.g., lidocaine or bupivacaine) into the anomalous joint to confirm that the pseudo-articulation is a primary source of the patient's pain [11,13]. The LSTV is considered to be the culprit if the injection leads to a significant reduction of their pain symptoms [1,8]. This relief tends to be short-lived where patients anecdotally report relief ranging from a few minutes to a couple of hours. A negative result after the injection indicates that the LSTV is likely not the pain generator [8]. 

Challenges leading to delayed diagnosis of Bertolotti’s syndrome

Large Variance in Age of Symptom Onset

Patients with BSy tend to present with chronic low back pain that started early in life. Some patients have been diagnosed as early as mid-to-late teens [31]. A recent retrospective cohort study of 150 individuals by Jenkins et al. showed that the average age of the cohort was 49 years old [8]. This large variance in age of symptom onset contributes to the delays in patients getting an accurate diagnosis.

Heterogeneity in Presentation of Symptoms

The primary symptom observed in the majority of patients is chronic low back pain [35]. Besides the low back pain, the L5 nerve root can be compressed between the enlarged transverse process and the sacral ala, a condition called far-out syndrome [14,15]. Furthermore, patients can experience pain localized to the gluteal region, hips, and groin area [35]. Due to this variety in the presentation of symptoms, patients face delays in getting an accurate diagnosis. 

Presence of "Collateral Damage"

Individuals with BSy often present with a highly variable constellation of symptoms. This clinical inconsistency is largely driven by the "collateral damage" resulting from the LSTV's abnormal biomechanics. The limited mobility of the LSTV transfers excessive mechanical stress to adjacent structures which can lead to secondary spinal pathologies. Such pathologies include a hypermobile segment immediately above the LSTV, leading to accelerating adjacent disc degeneration and disc herniations [8,11,20]. Furthermore, the clinical picture is often complicated by comorbid congenital conditions such as hEDS, which further compromises spinal stability. Despite this complex and varied presentation, certain mechanical pain triggers remain consistent across the BSy patient population. Symptoms are characteristically exacerbated by physical loading and movement, namely lumbar flexion and axial rotation [6,36]. Additionally, patients frequently report significant discomfort aggravated by prolonged sitting and specific sleeping positions.

All of these factors, i.e., variance in symptom onset, heterogeneity of symptom presentation, and presence of other spine conditions, all contribute to significant delays in getting a diagnosis. In fact, a recent systematic review by Zhu et al. showed that diagnosis of patients ranges from two months up to 16 years with a mean duration of around 41 months [35].

Treatment cascade for Bertolotti’s syndrome

Different studies have proposed a treatment cascade that begins with the least invasive options and progresses to more involved procedures [3,11,31]. Here, we aggregate the treatment cascade into three steps: conservative therapy, interventional therapy, and surgery. 

Step 1: Conservative Therapy

This step follows an integrative approach that utilizes pharmacotherapeutics, activity modification, and physical therapy. Conservative approaches have had varying degrees of success in mitigating pain symptoms and delaying the need for surgical intervention [1,3]. Nevertheless, it is critical that all conservative therapeutic approaches are explored before considering surgery.

Pharmacotherapeutics: Patients with BSy experience pain due to the inflammation caused by the arthritic pseudo-articulation or through secondary effects of the LSTV [6,15,20]. These symptoms can be mitigated using non-steroidal anti-inflammatory drugs (NSAIDs) such as naproxen, meloxicam and ibuprofen. Meloxicam is preferred for long-term management of symptoms over naproxen given its selectivity in its mechanism of action as well as lower risk of causing ulcers or renal side effects [37,38]. In patients presenting with severe muscle spasms in response to pain, a muscle relaxant may also be prescribed. 

Furthermore, BSy patients often experience neuropathic pain resulting from either mechanical nerve compression or chemical irritation secondary to local inflammation [3,11,35,39]. Pharmacological interventions such as gabapentinoids (e.g., gabapentin) and duloxetine are frequently employed to mitigate these symptoms [1,39]. Although evidence regarding their efficacy specifically within BSy populations remains limited, their therapeutic potential is supported by data in their use to treat rheumatological conditions, such as fibromyalgia, which present with comparable neurological symptoms [40]. More recently, low-dose naltrexone (LDN) has emerged as an effective option for managing neuropathic pain [41]. It has been shown to offer a relatively mild side-effect profile and significant symptomatic relief, often resulting in lower discontinuation rates compared to traditional neuropathic agents such as duloxetine and gabapentin [42,43].

As noted, there is currently no standardized pharmacotherapeutic protocol for BSy. Consequently, physicians play a pivotal role in helping patients navigate their longitudinal pain journey, identifying the therapeutic agents that offer optimal symptomatic relief while minimizing the adverse effects that contribute to treatment discontinuation. Although a comprehensive analysis of effective dosages, comparative molecular mechanisms, or the relative tolerability of these drugs is vital for developing a robust conservative therapy protocol, such an evaluation is beyond the scope of this review.

Occupational therapy: Providing guidance to patients on how to modify daily activities to reduce strain and promote energy conservation through pacing can be an effective way to reduce pain [44]. These recommendations should include activity modification to avoid movements that provoke pain as well as using adaptive equipment (e.g., reacher or shoe horn) to maintain independence in conducting daily activities.

Physical therapy: Patients with BSy have altered biomechanics due to the LSTV. These changes include hypermobility at adjacent segments, core instability, and asymmetric muscle tonicity in the lower back [10,12]. To devise the best treatment approach that is specific to each patient, a physical therapist should perform a thorough examination. The treatment protocol should aim to improve core stability, manage pain levels, and teach the patient ways to safely perform daily tasks without increasing pain levels. 

Step 2: Interventional Therapy

If the conservative therapeutic approaches fail to provide adequate relief, interventional pain management techniques such as corticosteroid injections and radiofrequency ablation (RFA) become important second-line therapy for patients. Due to the paucity of published research on the use of injections or RFA for treating BSy, we have relied on case reports and small-scale studies in our analysis. 

Corticosteroid injection to the pseudo-articulation can be administered in order to alleviate inflammation [14]. This, coupled with conservative therapies outlined above, can provide lasting pain relief for patients. In some cases, corticosteroid injections only provide temporary relief requiring periodic re-administration [1]. 

In some patients, L4/L5 facet joint RFA has been used to successfully reduce pain at these levels [45,46]. While RFA is a conventional procedure used to treat pain emanating from the facet joints generally and is not a specialized treatment for BSy patients only, its specific utility for this population requires further evaluation. Based on our findings, there is no consensus on the relative efficacy of the different types of RFA techniques: thermal, cooled, or pulsed RFA. This further emphasizes the need for further clinical research to identify interventional pain management techniques that would be effective for BSy patients.

Step 3: Surgery

When a comprehensive course of conservative and interventional management fails to provide lasting relief for a patient with debilitating pain from BSy, surgical intervention becomes a viable option. Two surgical approaches are commonly used to directly address the direct effects of LSTV: resection (transverse processectomy) or fusion. 

Resection surgery aims to directly address the source of mechanical pain by surgically removing or "shaving down" the enlarged transverse process [20,39]. This eliminates the bone-on-bone contact responsible for mechanical grinding, inflammation, and the formation of osteophytes [3]. Given recent advances, resection surgery utilizes minimally invasive surgical techniques using microscopic tubular resection or endoscopic resection [20,39]. These utilize smaller incisions and specialized instruments to minimize damage to the surrounding muscles and tissues [20]. Resection surgery is typically performed in patients with minimal co-morbidities; namely, patients that do not have spinal stenosis, significant degeneration of adjacent segments, disc herniation, nor the presence of other congenital conditions associated with BSy.

Fusion surgery aims to completely eliminate motion at the painful segment by creating a solid bridge of bone, effectively making the transitional vertebra a permanent part of the sacral base. This is typically achieved with posterolateral fusion using bone graft material and stabilization with pedicle screws and rods [13]. Bone morphogenic protein-2 (rhBMP-2) is sometimes used off-label to infuse the bone graft material in order to facilitate fusion [13]. 

Resection Versus Fusion

There is likely no “one size fits all” surgical approach for BSy patients. The right surgical approach must take into account a multitude of factors such as the type of LSTV, patient co-morbidities, age of patient, surgical history, and more. Resection surgery holds appeal to many patients given the minimally invasive approach and the fast recovery time. However, it does come with the risk of introducing further instability [13]. The enlarged transverse processes of the LSTV provide some degree of stability to the lumbosacral junction. Removing this structure without addressing underlying instability can lead to poor long-term outcomes [13]. 

On the other hand, fusion is generally considered the more appropriate choice for patients who have evidence of spinal instability, advanced degenerative disc disease at the adjacent level, spondylolisthesis, or in cases where a simple resection is deemed likely to cause further spine instability [3,11,20,39]. However, fusion surgery can come with its own set of risks including accelerating degeneration of segments above the fusion site (i.e., adjacent segment disease) and hardware-related issues [14,20].

A recent retrospective cohort study of 150 individuals by Jenkins et al. has taken steps to develop a systematic framework that uses the anatomy of the LSTV for determining the appropriate surgical approach [13]. Patient outcomes were tracked up to 2 years post-surgery. Based on these results, the authors offer surgical recommendations for the different classes of LSTV: resection surgery for Type 1 patients, bilateral fusion surgery for Type 2 patients, and unilateral fusion for Type 4 patients [13]. To our knowledge, this is the first study to attempt to associate a surgical approach that yields the best outcomes based on a patient's LSTV anatomy. It lays a strong foundation upon which further studies can build upon to develop a robust clinical protocol to select the appropriate surgical technique that yields the best outcomes for the patient.

Areas for further investigation

Despite significant advances in understanding and managing BSy, there are several critical areas that merit investigation.

Broad Adoption of a Unified LSTV Classification System

While the Jenkins classification offers a robust methodology that addresses the diagnostic limitations of the Castellvi classification, it has yet to be widely adopted across the medical field. Establishing a standardized nomenclature specific to BSy is critical for ensuring effective communication between diverse specialties, including radiology, neurology, pain management, and rehabilitation. A unified language is the first step toward systematically documenting patient presentations, which will eventually allow researchers to aggregate data and correlate specific symptom profiles with distinct LSTV classifications.

Development of Comprehensive Physical Therapy Guidelines

Although the principles of targeted physical therapy are well-established, there are no protocols tailored to BSy patients. This gap extends to surgical care where there are currently no standardized pre- or post-operative recovery frameworks for patients undergoing resection or fusion. Establishing and validating evidence-based protocols could significantly enhance non-operative outcomes, potentially reducing or delaying the need for surgical intervention. Furthermore, robust rehabilitation strategies are essential to address post-surgical compensation patterns and improve long-term clinical results.

Clarity on Effective Surgical Techniques

There exists insufficient data in current literature regarding long-term surgical outcomes for BSy. To address this, it is imperative to have longitudinal cohort studies that monitor patients both pre- and post-operatively to accurately assess intervention effectiveness. Large-scale, long-term evaluations of patient outcomes are essential for developing evidence-based protocols that empower physicians to select the appropriate surgical approach tailored to a patient’s specific clinical profile. While Jenkins et al. [13] offered a foundational framework for systematizing surgical selection based on anatomy and symptoms, further validation is required before these findings can be universally adopted. The ultimate objective is to establish a rigorous clinical rubric that guides surgeons in determining the most appropriate surgical approach.

Establish a Global Patient Registry

The establishment of a global patient registry is critical for tracking the natural history of BSy beyond isolated surgical cohorts. Integrating Patient-Reported Outcome Measures (PROMs) into such a registry provides a quantifiable metric for patient burden that imaging alone fails to capture. This methodology has been instrumental in other rare disease contexts; for example, the use of registry-based PROMs in Barth syndrome research has successfully characterized the "diagnostic odyssey" and multi-systemic clinical burden, offering a clearer understanding of the condition's natural history [47]. For BSy, this data is vital for enhancing diagnostic rigor by correlating specific LSTV types with functional impairments. Ultimately, a robust dataset of PROMs can demonstrate the long-term efficacy of various interventions, guiding the development of more effective, patient-centered treatment protocols.

## Conclusions

Misdiagnosis and diagnostic delays represent the major challenges for patients living with BSy. This often leads to years of unmanaged chronic low back pain and ineffective treatments. While an LSTV is relatively common in the general population, the challenge is distinguishing an incidental radiological finding from the LSTV being the true pain generator. This narrative review attempts to address this challenge by consolidating the latest findings to provide a comprehensive framework detailing the condition’s origins, diagnostic protocols, and treatment options. Ultimately, broadening the awareness of this often overlooked condition is important to reduce diagnostic delays and deliver more effective care to patients.
